# A Rare Case of Yellow Urticaria Developing During Blood Transfusion

**DOI:** 10.7759/cureus.105814

**Published:** 2026-03-25

**Authors:** Lana T Alsemairi, Azhar Ahmed, Abdurrahman I Ibrahim, Mustafa Zaytuni, Kholod H Aljohani

**Affiliations:** 1 Dermatology, Uhud General Hospital, Madinah, SAU; 2 Dermatology, King Faisal Specialist Hospital and Research Centre, Madinah, SAU; 3 Medicine, Taibah University, Madinah, SAU

**Keywords:** adverse transfusion reaction, anemia and hyperbilirubinemia, blood transfusion reaction, urticaria, yellow urticaria

## Abstract

Yellow urticaria is a rare clinical variant of urticaria characterized by yellowish wheals, typically associated with hyperbilirubinemia. Its occurrence as a transfusion-related reaction is uncommon and may mimic more serious transfusion complications. We report the case of a 15-year-old Saudi female patient who presented with prolonged vaginal bleeding and severe iron deficiency anemia requiring packed red blood cell transfusion. Approximately 15 minutes after transfusion initiation, she developed pruritic yellowish urticarial wheals over the trunk and extremities, without angioedema or hemodynamic instability. Laboratory evaluation revealed mild hyperbilirubinemia, while direct and indirect Coombs tests were negative, and no clinical or laboratory evidence suggestive of hemolysis was identified. The transfusion was temporarily interrupted, and the patient was treated with antihistamines, corticosteroids, and epinephrine, leading to complete resolution of the lesions within 24 hours. Transfusion was later resumed without recurrence. This case highlights transfusion-related yellow urticaria as a benign but rare reaction. Early recognition is essential to avoid misdiagnosis and unnecessary interruption of life-saving transfusion therapy.

## Introduction

Urticaria is a common dermatologic condition characterized by the sudden onset of transient, pruritic wheals resulting from mast cell activation and the release of inflammatory mediators, particularly histamine, leading to increased vascular permeability and dermal edema [1]. Clinically, urticarial wheals are typically erythematous or skin-colored, well-circumscribed, and resolve spontaneously within hours without residual skin changes.

Although the classic appearance of urticaria is well recognized, several atypical variants have been described. Yellow urticaria represents a rare and unusual form, in which wheals demonstrate a distinctive yellow to yellow-orange discoloration rather than the conventional erythematous hue. This rare presentation has been consistently associated with elevated serum bilirubin levels, most commonly in patients with underlying hepatobiliary disease or systemic hyperbilirubinemia [2].

The pathophysiology of yellow urticaria is believed to involve increased capillary permeability during urticarial reactions, allowing circulating bilirubin to extravasate from the intravascular compartment into the dermis. Accumulation of bilirubin within the edematous wheals imparts the characteristic yellow coloration, which remains localized to the urticarial lesions and differs from the diffuse skin discoloration observed in generalized jaundice [3].

Despite its striking clinical appearance, yellow urticaria is not considered a distinct disease entity, but rather a cutaneous manifestation secondary to systemic biochemical abnormalities, particularly hyperbilirubinemia [3]. To date, fewer than 10 cases of yellow urticaria have been reported in the literature, highlighting the rarity of this condition. Published reports have documented yellow urticaria in association with chronic liver disease, alcoholic cirrhosis, biliary obstruction, gallstone disease, and other hepatobiliary disorders leading to elevated bilirubin levels [2,4].

In some cases, yellow urticaria has served as an important diagnostic clue, prompting further evaluation and leading to the identification of previously unrecognized systemic or hepatobiliary pathology, including biliary pancreatitis [2]. Additionally, yellow urticaria has been reported as a presenting manifestation of underlying autoimmune overlap syndromes, further emphasizing its potential role as a marker of systemic disease [5].

Systemic triggers have also been implicated in the development of yellow urticaria, particularly in susceptible patients with pre-existing liver dysfunction. Notably, cases have been described following blood transfusion, where the urticarial reaction, combined with elevated bilirubin levels, resulted in the characteristic yellow wheals [6].

Due to its atypical appearance, yellow urticaria may be misdiagnosed as other causes of yellow skin discoloration, including xanthomas, carotenoderma, or jaundice-related dermatoses. Awareness of this rare entity is therefore essential to ensure appropriate diagnostic evaluation and to avoid overlooking potentially significant underlying systemic disease [6]. Reporting additional cases contributes to a better understanding of its clinical spectrum, pathogenesis, and diagnostic significance.

## Case presentation

A previously healthy 15-year-old Saudi female patient presented to the emergency department with a complaint of persistent vaginal bleeding for approximately one month. On presentation, she was fully conscious, oriented, and hemodynamically stable. Her vital signs were within normal limits, including a blood pressure of 122/60 mmHg, heart rate of 81 beats per minute, respiratory rate of 18 breaths per minute, temperature of 36.5 °C, and oxygen saturation of 99% on room air.

The patient reported regular menstrual cycles since menarche at the age of 12 years, with no prior history of menstrual irregularity until the current episode. She denied the use of hormonal therapy and had no history of gynecological procedures. She reported a previous episode of severe anemia approximately one year prior that required blood transfusion. There was no history of chronic medical illness, known drug allergies, autoimmune disease, or previous documented transfusion reactions. She was not taking any medications at the time of admission.

**Figure 1:**
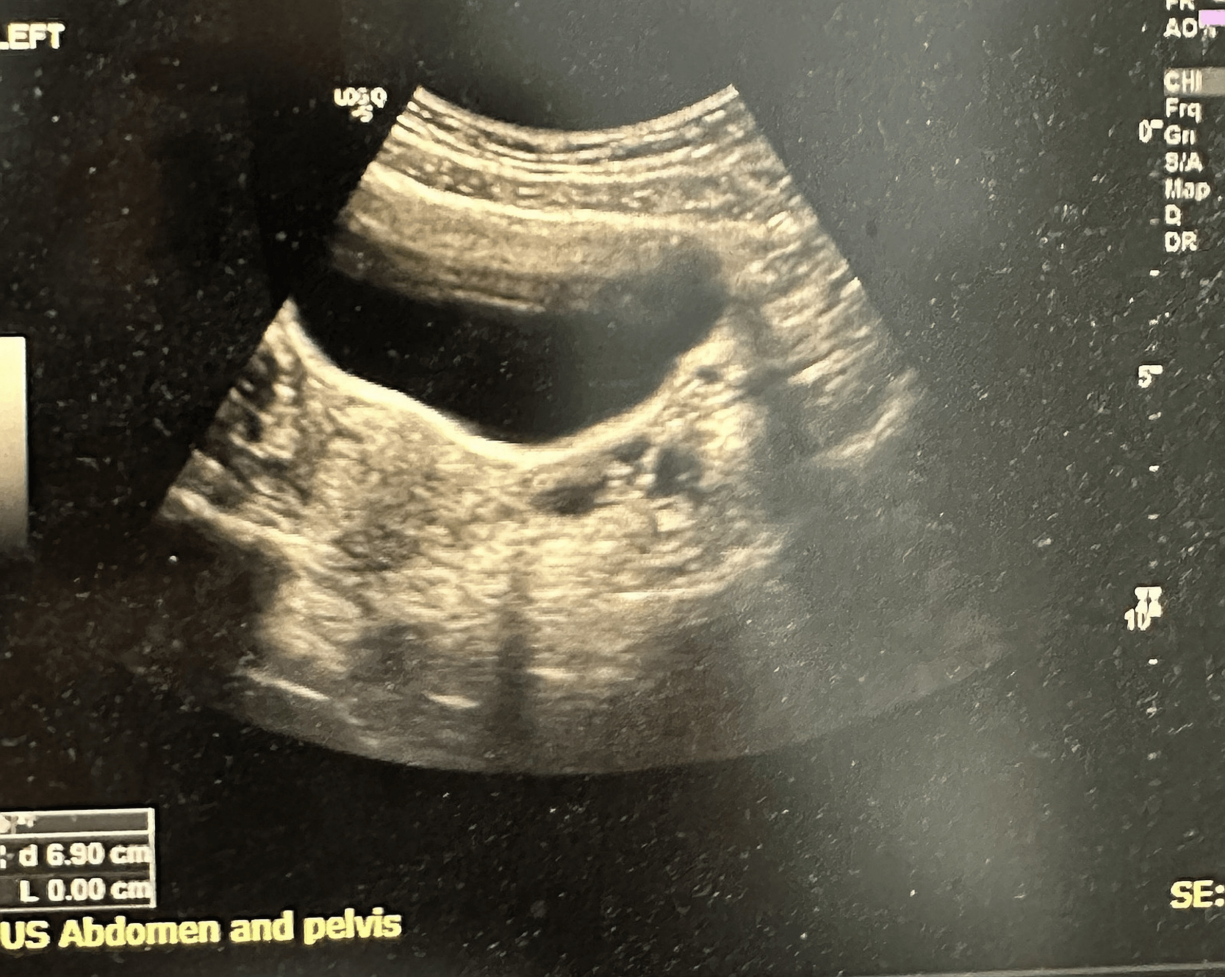
Pelvic ultrasonography demonstrating a right adnexal cyst measuring approximately 4.4 × 3.1 cm.

On physical examination, the patient appeared pale but comfortable. Abdominal examination revealed a soft, non-tender abdomen with no palpable organomegaly. Pelvic ultrasonography demonstrated an anteverted uterus with an endometrial thickness of 0.9 cm and a right adnexal cyst measuring approximately 4.4 × 3.1 cm. Both ovaries were otherwise unremarkable, with no focal uterine lesions or pelvic free fluid (Figure 1).

Initial laboratory investigations revealed severe microcytic hypochromic anemia (low mean corpuscular volume (MCV), mean corpuscular hemoglobin (MCH), and mean corpuscular hemoglobin concentration (MCHC) with elevated red cell distribution width (RDW)), strongly suggestive of iron deficiency anemia in the context of prolonged vaginal bleeding. Liver function tests showed elevated transaminases with mild hyperbilirubinemia, which may reflect transient hepatic stress related to severe anemia and the acute transfusion reaction rather than primary liver disease. Platelet count, white blood cell count, renal function, electrolytes, and coagulation profile were within normal ranges. Detailed laboratory findings are summarized in Table 1.

**Table 1:** Summary of laboratory investigations. MCV: mean corpuscular volume, MCH: mean corpuscular hemoglobin, MCHC: mean corpuscular hemoglobin concentration, RDW: red cell distribution width, ALT: alanine aminotransferase, AST: aspartate aminotransferase, BUN: blood urea nitrogen, PT: prothrombin time, INR: international normalized ratio, PTT: partial thromboplastin time.

Laboratory parameter	Result	Reference range
Hemoglobin	5.6 g/dL	11.5-16.5 g/dL
Hematocrit	20.4%	37%-47%
White blood cells	7.60 × 10⁹/L	4.5-10 × 10⁹/L
Platelet count	279 × 10⁹/L	150-410 × 10⁹/L
MCV	71.36 fL	76-96 fL
MCH	20.60 pg	27-32 pg
MCHC	28.86 g/dL	30-35 g/dL
RDW	18.21%	11.5%-14.5%
ALT	104 U/L	0-45 U/L
AST	144 U/L	0-35 U/L
Total bilirubin	23.9 µmol/L	0-18.7 µmol/L
Direct bilirubin	9.4 µmol/L	0-3.4 µmol/L
Creatinine	58 µmol/L	80-115 µmol/L
BUN	2.8 mmol/L	2.1-7.1 mmol/L
Sodium	141 mmol/L	136-145 mmol/L
Potassium	4.54 mmol/L	3.5-5.1 mmol/L
Phosphorus	1.32 mmol/L	0.8-1.4 mmol/L
Magnesium	0.83 mmol/L	0.6-1.07 mmol/L
Calcium	2.34 mmol/L	2.15-2.65 mmol/L
Albumin	42 g/L	35-52 g/L
Alkaline phosphatase	55 U/L	39-117 U/L
PT	12.5 s	11-15 s
INR	1.09	0.9-1.2
PTT	24.1 s	25-40 s

**Figure 2:**
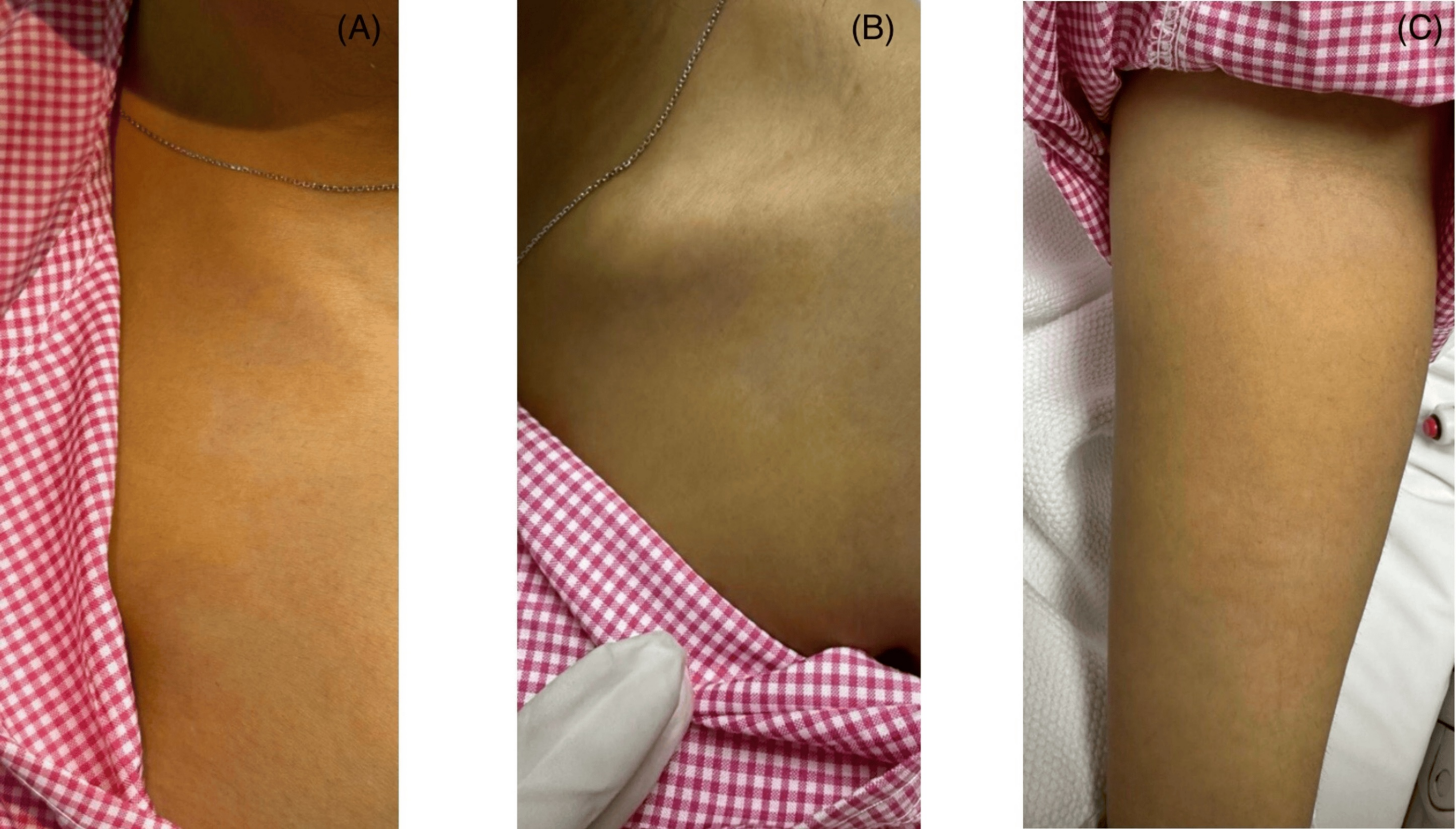
Lesions that developed approximately 15 minutes after initiation of packed red blood cell transfusion, without associated angioedema or mucosal involvement. (A) Multiple well-demarcated, raised yellowish urticarial wheals over the anterior trunk. (B) Localized yellow edematous wheals involving the upper chest and supraclavicular region, with discoloration confined to the lesions and sparing the surrounding skin. (C) Similar well-circumscribed yellow urticarial wheals distributed over one upper extremity (arm).

Given the severity of anemia, an urgent packed red blood cell transfusion was arranged, and the patient was admitted to the intensive care unit (ICU) for close monitoring. Approximately 15 minutes after initiation of the first unit of packed red blood cells, the patient developed sudden-onset pruritic, raised wheals with a distinctive yellowish discoloration involving the trunk and extremities (Figure 2). There was no associated angioedema, mucosal involvement, respiratory distress, hypotension, or altered level of consciousness. Vital signs remained stable throughout the episode.

The transfusion was immediately interrupted, and a transfusion reaction workup was initiated. Direct and indirect Coombs tests were negative, effectively excluding hemolytic transfusion reaction. The transfused blood unit was confirmed to be type O-positive, cross-matched, and screened negative for infectious markers prior to administration.

A dermatology consultation was obtained. Dermoscopic examination was not performed. Clinical examination confirmed the presence of yellowish urticarial wheals over the trunk and extremities, with no evidence of angioedema or airway compromise. The dermatology team concluded that the findings were consistent with urticaria following blood transfusion, possibly related to underlying hyperbilirubinemia. Differential diagnoses included hemolytic transfusion reaction, drug-induced urticaria, and infectious hepatitis. Hemolysis was ruled out by negative Coombs testing and clinical stability, drug-induced urticaria was unlikely due to the absence of recent medication exposure, and infectious hepatitis was considered unlikely given the lack of systemic symptoms and only mild liver enzyme elevation. Other dermatologic conditions that may produce yellowish skin lesions, such as xanthomas and carotenoderma, were also considered. However, these conditions typically present with persistent yellow discoloration rather than acute pruritic wheals and were therefore considered unlikely in this case.

The patient was treated with intravenous methylprednisolone 125 mg, oral cetirizine 10 mg, and intramuscular epinephrine 0.3 mg after initial refusal. Intravenous Ringer’s lactate was administered at a rate of 100 mL/hour. The urticarial lesions improved rapidly and resolved completely within 24 hours. The patient remained under close observation in the ICU, after which blood transfusion was cautiously resumed without recurrence of symptoms.

Following clinical stabilization and improvement of hemoglobin levels to above 8 g/dL, the patient was transferred from the ICU to the general ward. She was discharged in stable condition with oral ferrous sulfate 200 mg daily, tranexamic acid 500 mg twice daily for five days, and dydrogesterone 10 mg daily for seven days, to be continued cyclically for three months. She was advised to maintain an iron-rich diet and attend dermatology and gynecology follow-up.

At one-week follow-up, the patient was clinically well, afebrile, and free of skin lesions, with no recurrence of urticaria. Repeat laboratory investigations demonstrated improvement in hemoglobin level, with bilirubin and liver enzyme levels trending toward normal values.

## Discussion

Yellow urticaria is a rare chromatic variant of urticaria in which wheals exhibit a yellowish discoloration instead of the typical erythematous appearance. This phenomenon is thought to result from the deposition of bilirubin within the dermal edema of urticarial lesions during episodes of increased vascular permeability [3]. Most reported cases have occurred in patients with underlying hyperbilirubinemia related to hepatobiliary disease, making this presentation uncommon and often underrecognized. In the present case, the patient developed pruritic yellowish urticarial wheals approximately 15 minutes after initiation of packed red blood cell transfusion. The close temporal relationship strongly suggests a transfusion-related allergic reaction, which is a recognized but generally benign complication of blood transfusion [1]. However, the distinctive yellow coloration of the wheals represents an atypical feature that differentiates this case from conventional transfusion-related urticaria. The most plausible pathophysiological explanation involves the coexistence of two mechanisms: an acute urticarial reaction triggered by transfusion-related immune pathways and preexisting mild hyperbilirubinemia that allowed bilirubin to extravasate into the dermis during increased capillary permeability [3,6]. Although the degree of hyperbilirubinemia in our patient was mild, similar levels have been reported in previous cases of yellow urticaria and may be sufficient to produce localized yellow discoloration when bilirubin extravasates into urticarial wheals. Unlike generalized jaundice, bilirubin deposition in yellow urticaria is localized to the edematous wheals, resulting in sharply demarcated yellow lesions [6].

This presentation may raise concern for more serious transfusion reactions, particularly hemolytic transfusion reactions, which can also be associated with elevated bilirubin levels. In our patient, hemolysis was effectively excluded by negative direct and indirect Coombs testing, stable vital signs, and the absence of systemic manifestations, supporting a diagnosis of transfusion-related yellow urticaria rather than a hemolytic process. Recognition of this distinction is clinically important, as misdiagnosis may lead to unnecessary cessation of transfusion or extensive diagnostic evaluation.

Previously reported cases of yellow urticaria have predominantly involved adult patients with chronic liver disease, biliary pathology, or autoimmune conditions associated with hyperbilirubinemia [4,5,7,8] (Table 2). Koumaki et al. described yellow urticaria in a patient with alcohol-related liver cirrhosis, while Toujani et al. reported yellow urticaria as a manifestation of an autoimmune overlap syndrome [4,5]. In contrast, the present case is notable for occurring in an adolescent patient without known chronic liver disease, highlighting that even mild hyperbilirubinemia may predispose to this rare manifestation when combined with an acute urticarial trigger such as blood transfusion [4]. Management of yellow urticaria does not differ substantially from that of conventional urticaria. Antihistamines remain the cornerstone of treatment, with corticosteroids and epinephrine reserved for more pronounced reactions or systemic involvement [1]. In our case, prompt intervention resulted in complete resolution of skin lesions within 24 hours, and blood transfusion was later safely resumed without recurrence, further supporting the benign nature of this reaction when appropriately recognized and managed. This case emphasizes the importance of clinician awareness of yellow urticaria as a rare but benign variant of transfusion-related urticaria and highlights the need to differentiate it from severe transfusion reactions such as hemolysis and anaphylaxis. Awareness of this entity may prevent misinterpretation as a hemolytic transfusion reaction, avoid unnecessary interruption of life-saving transfusion therapy, and guide appropriate reassurance and management.

**Table 2:** Summary of previously reported cases of yellow urticaria.

Associated comorbidities/notes	Gender	Patient age	Author (year)
EAACI/GA²LEN/EDF/WAO guideline for the definition, classification, diagnosis, and management of urticaria	Not applicable	Not applicable	Zuberbier et al. (2018) [1]
Biliary pancreatitis with hyperbilirubinemia presenting with yellow urticaria	Female	82 years	Nava Cruz et al. (2017) [2]
Systematic review of previously reported cases of yellow urticaria	Not applicable	Not applicable	Kulthanan et al. (2023) [3]
Alcohol-related liver cirrhosis with jaundice	Male	61 years	Koumaki et al. (2021) [4]
Autoimmune hepatitis with primary biliary cholangitis overlap syndrome	Female	33 years	Toujani et al. (2023) [5]
Biliary obstruction due to gallstones with hyperbilirubinemia	Male	24 years	Almutairi et al. (2025) [6]
Liver disease following blood transfusion with hyperbilirubinemia	Male	67 years	Hardy et al. (2022) [7]
Narrative review of urticaria differential diagnosis	Not applicable	Not applicable	Schettini et al. (2023) [8]

## Conclusions

Yellow urticaria is a rare and underrecognized manifestation of acute urticaria, often associated with underlying hyperbilirubinemia. This case emphasizes that yellow urticarial lesions occurring during blood transfusion do not necessarily indicate a severe transfusion reaction or hemolysis. Prompt recognition, exclusion of life-threatening causes, and appropriate symptomatic management can lead to rapid resolution without compromising essential transfusion therapy. Increased awareness of this entity may help clinicians avoid unnecessary diagnostic interventions and premature discontinuation of blood transfusions.

## References

[1] Zuberbier T, Aberer W, Asero R (2018). The EAACI/GA²LEN/EDF/WAO guideline for the definition, classification, diagnosis and management of urticaria. Allergy.

[2] Nava Cruz EM, Castanedo-Cázares JP, Hernández Blanco DV, Torres-Álvarez B (2017). Yellow urticaria and biliary pancreatitis. JAAD Case Rep.

[3] Kulthanan K, Tanwandee T, Chularojanamontri L, Panjapakkul W, Saengthong-Aram P (2023). Yellow urticaria: a systematic review. J Asthma Allergy.

[4] Koumaki D, Demetriou G, Krasagakis K (2021). Yellow urticaria in a patient with alcohol-related liver cirrhosis and jaundice. Indian J Dermatol Venereol Leprol.

[5] Toujani S, El Ouni A, Mestiri S, Toujani E, Belhassen A, Meddeb Z, Bouslama K (2023). Yellow urticaria revealing overlap syndrome of autoimmune hepatitis and primary biliary cholangitis. Clin Case Rep.

[6] Almutairi M, Nagshabandi KN, Alsergani R, Alsalem S (2025). Yellow urticaria as a rare dermatological manifestation of biliary obstruction: a case report. Dermatol Reports.

[7] Hardy S, King R, Scholand S (2022). Yellow urticaria in a patient with liver disease following transfusion. Case Rep Clin Med.

[8] Schettini N, Corazza M, Schenetti C, Pacetti L, Borghi A (2023). Urticaria: a narrative overview of differential diagnosis. Biomedicines.

